# Supplementary material for: Abrupt onset of intensive human occupation 44,000 years ago on the threshold of Sahul
Source: Nat Commun. 2024 May 22;15:4193. doi: 10.1038/s41467-024-48395-x (PMC11111772; doi:10.1038/s41467-024-48395-x)
Supplement: Supplementary file 1 — Supplementary Information [file 41467_2024_48395_MOESM1_ESM.pdf]

Supplementary Information

Supplementary Figures

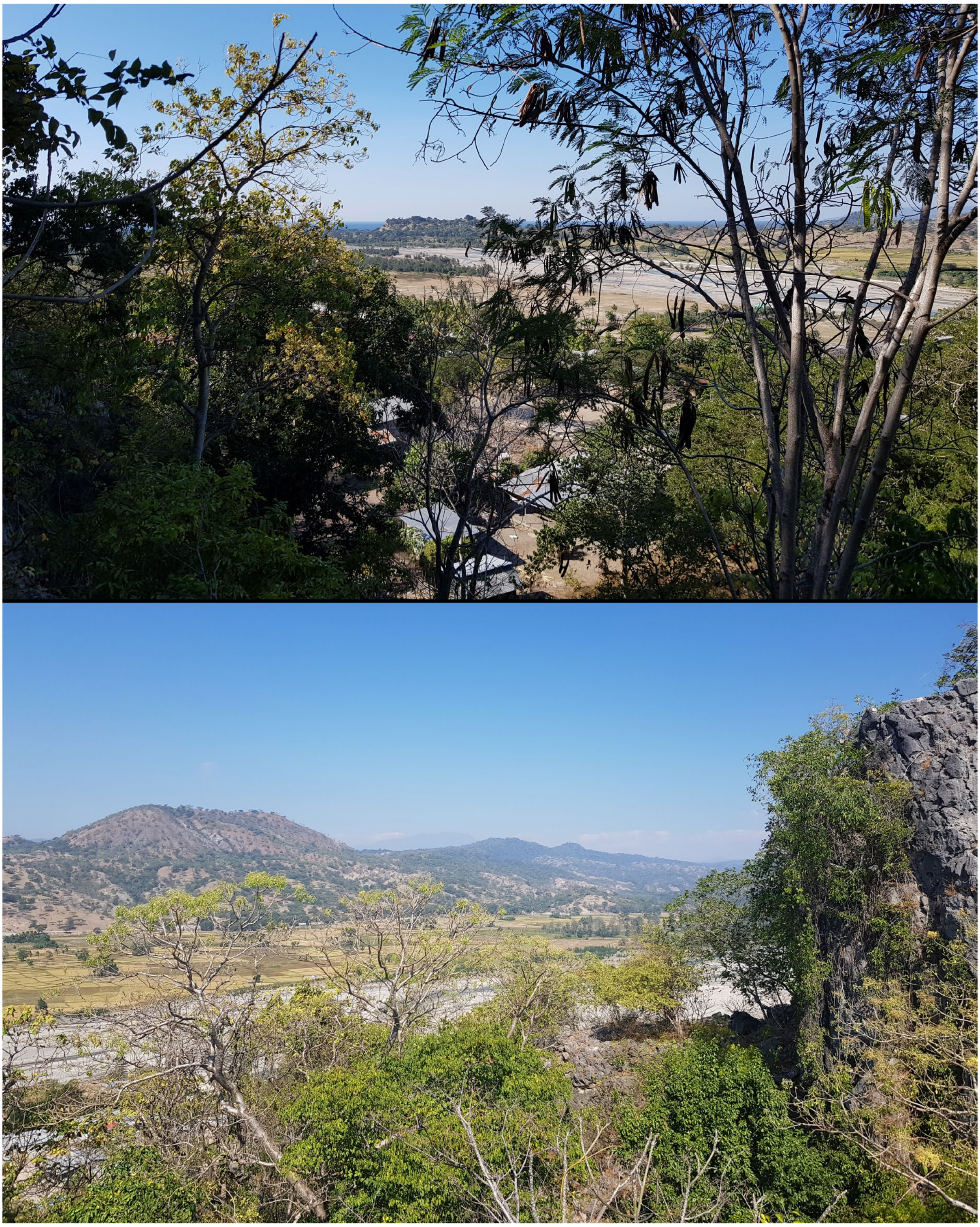

Supplementary Figure 1. *Views from above Laili rockshelter. Above - looking downstream along the Laleia river and out to sea. Below looking upstream along the Laleia river and into the mountains.*

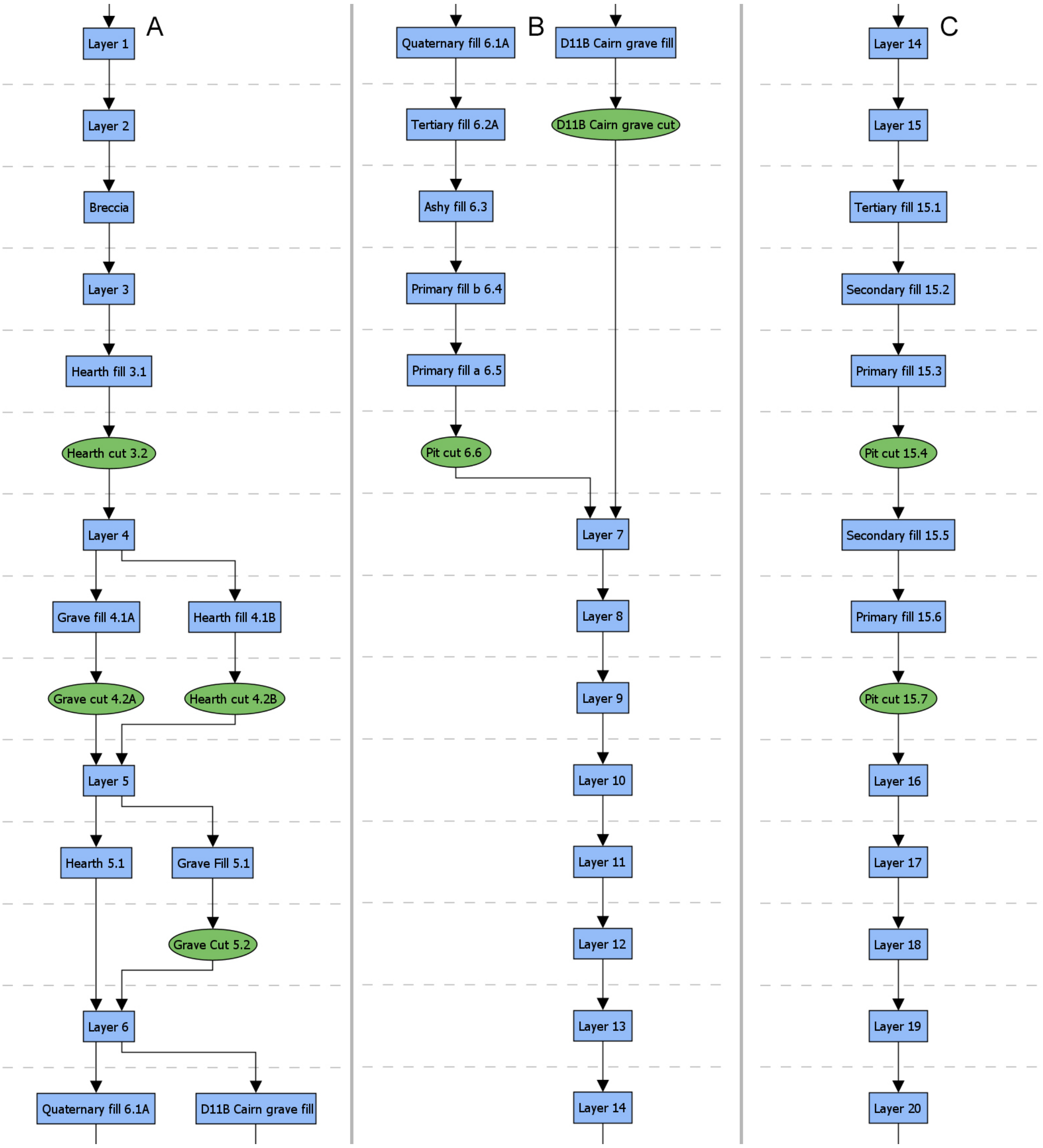

Supplementary Figure 2. *Harris matrix showing the relationship between the deposit layers, the cave wall breccia, as well as cut and fill features for the upper (A), middle (B), and lower (C) parts of the Laili sequence. Layers and fills are shown as blue rectangles, cuts are shown as green ellipses. Layer 20 is above the culturally sterile Layer 21.*

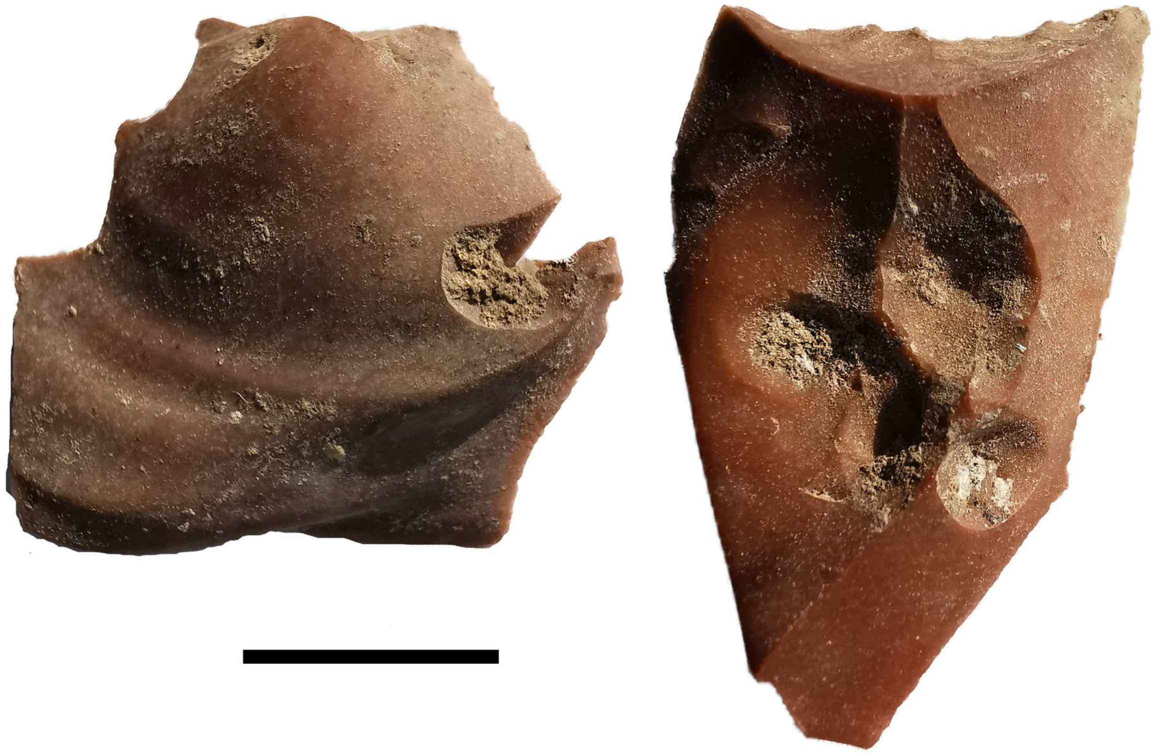

Supplementary Figure 3. *Lithics from the sondage showing intrusive ashy grey-brown sediment adhering to them in pot-lid cavities, distinct from the pale-yellow surrounding matrix. Scale bar is 1 cm.*

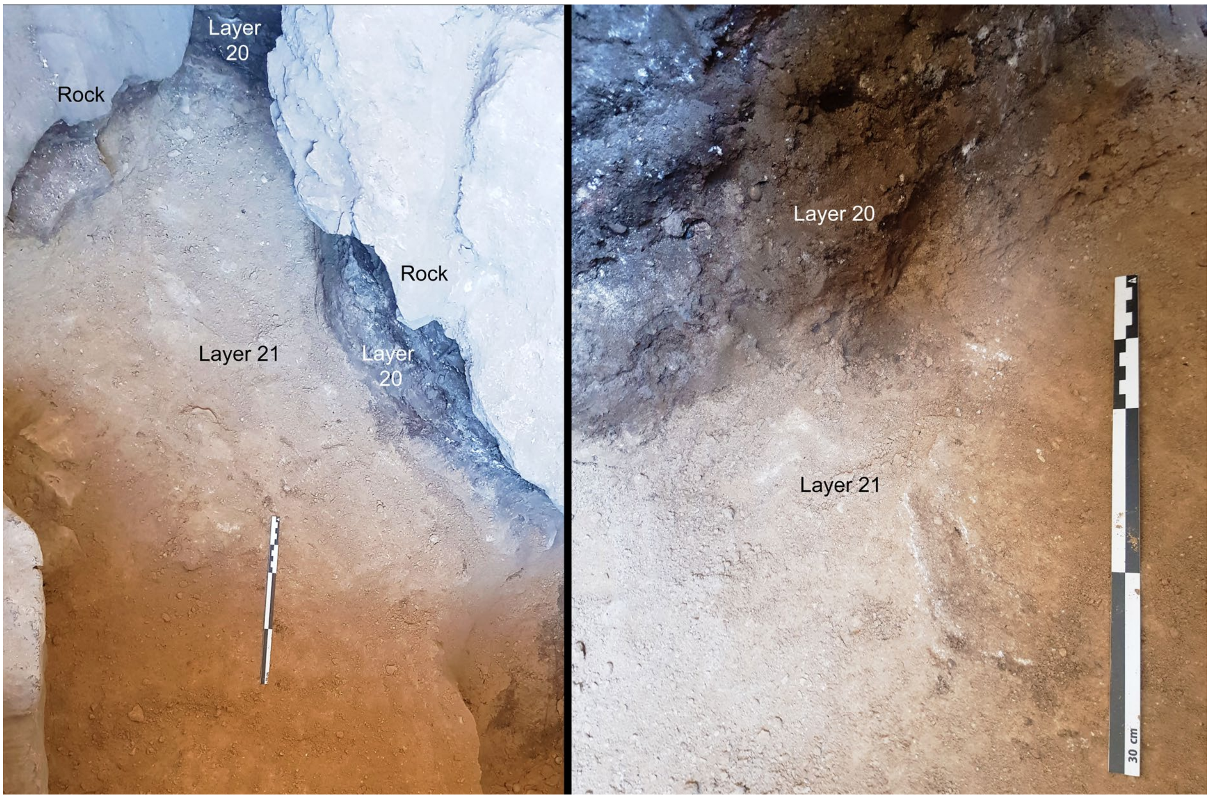

Supplementary Figure 4. *The contrast in colour at the macro-scale between culturally sterile Layer 21 exposed horizontally during excavation and the initial occupation of Layer 20 visible in the section wall. Scale bar is 30 cm.*

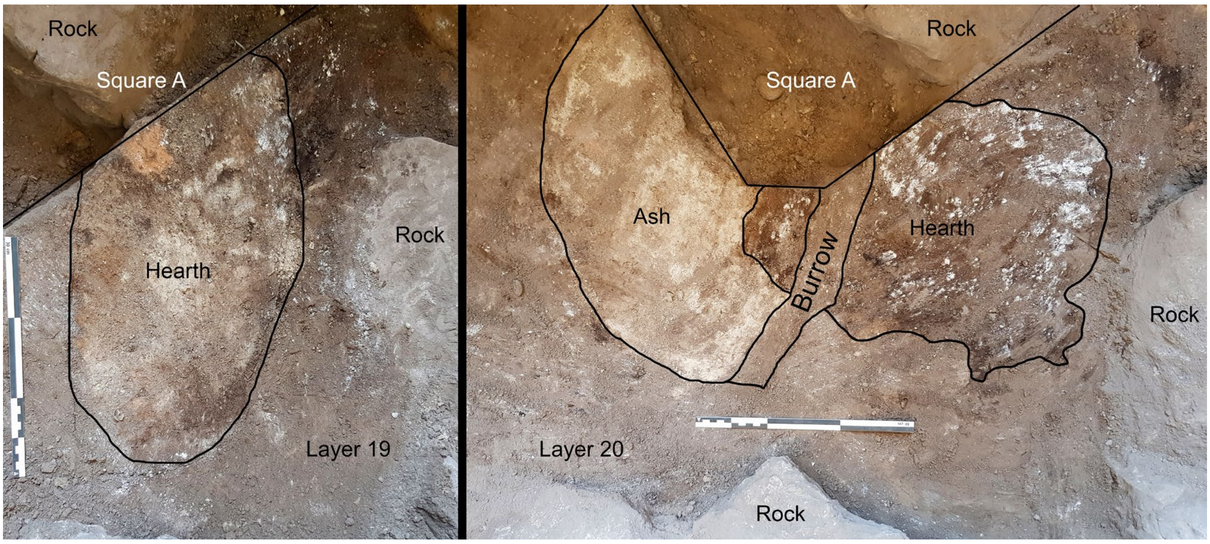

Supplementary Figure 5. *Horizontally discrete combustion features in Layers 19 and 20. Scale bar is 30 cm.*

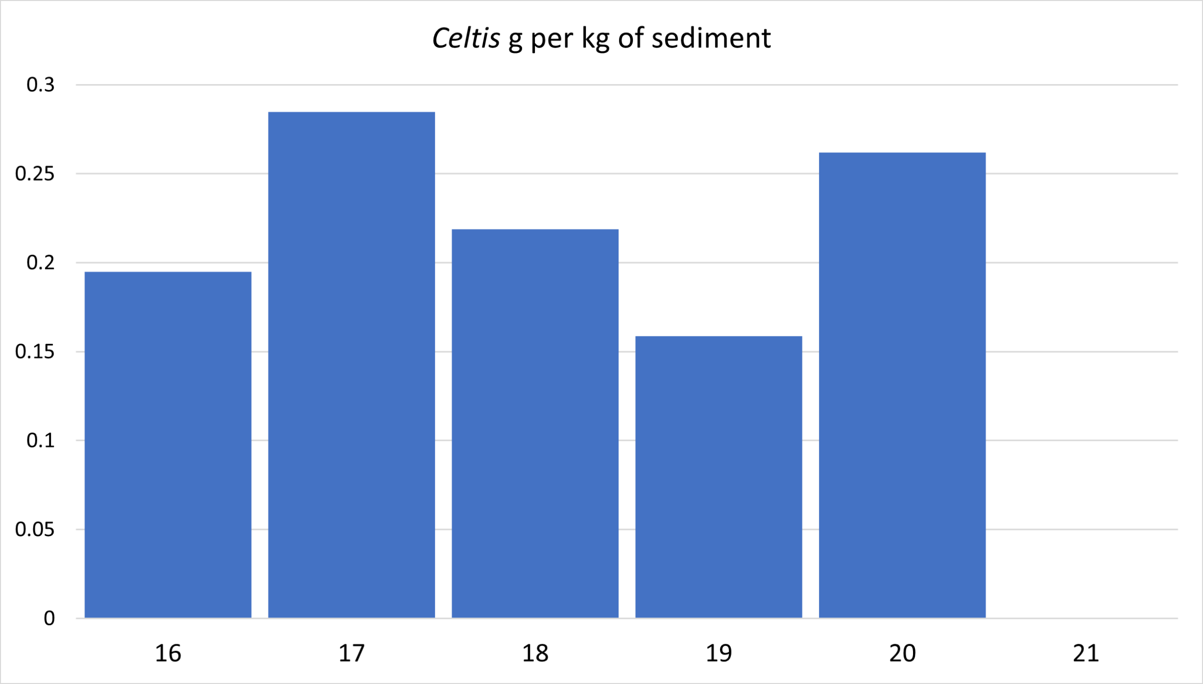

Supplementary Figure 6. *Celtis seeds per kg of sediment by layer. Source data are provided in the Source Data file.*

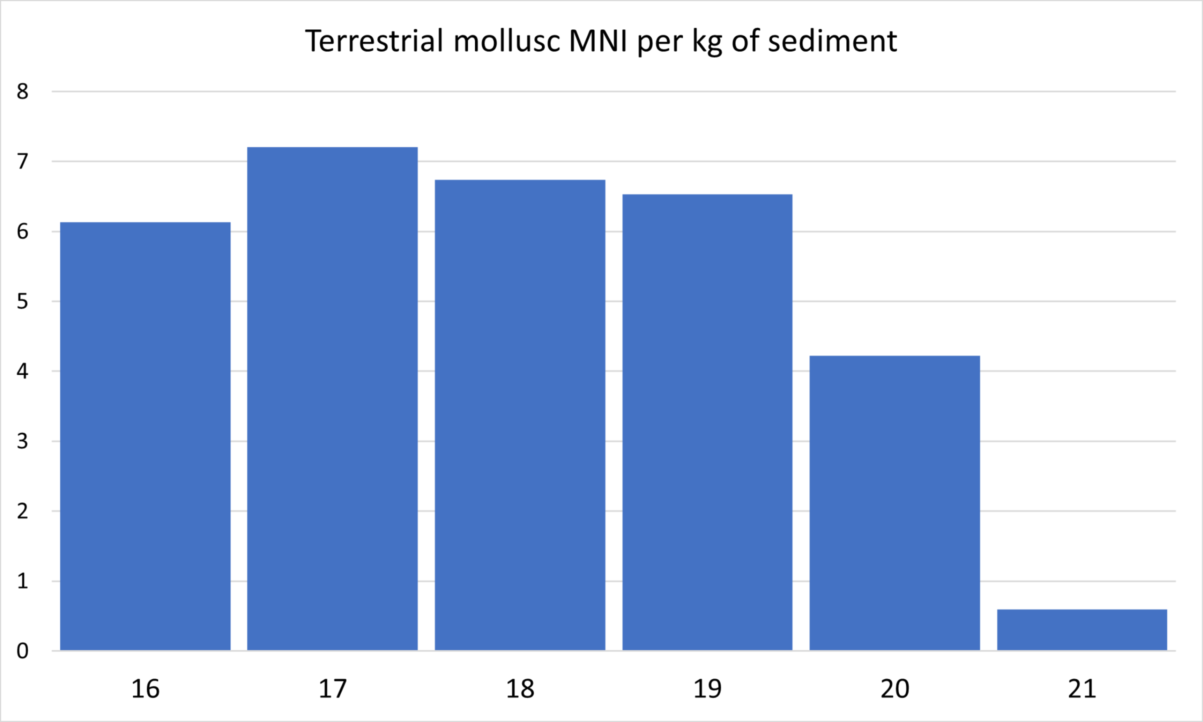

Supplementary Figure 7. *Terrestrial mollusc MNI per kg of sediment by layer. Source data are provided in the Source Data file.*

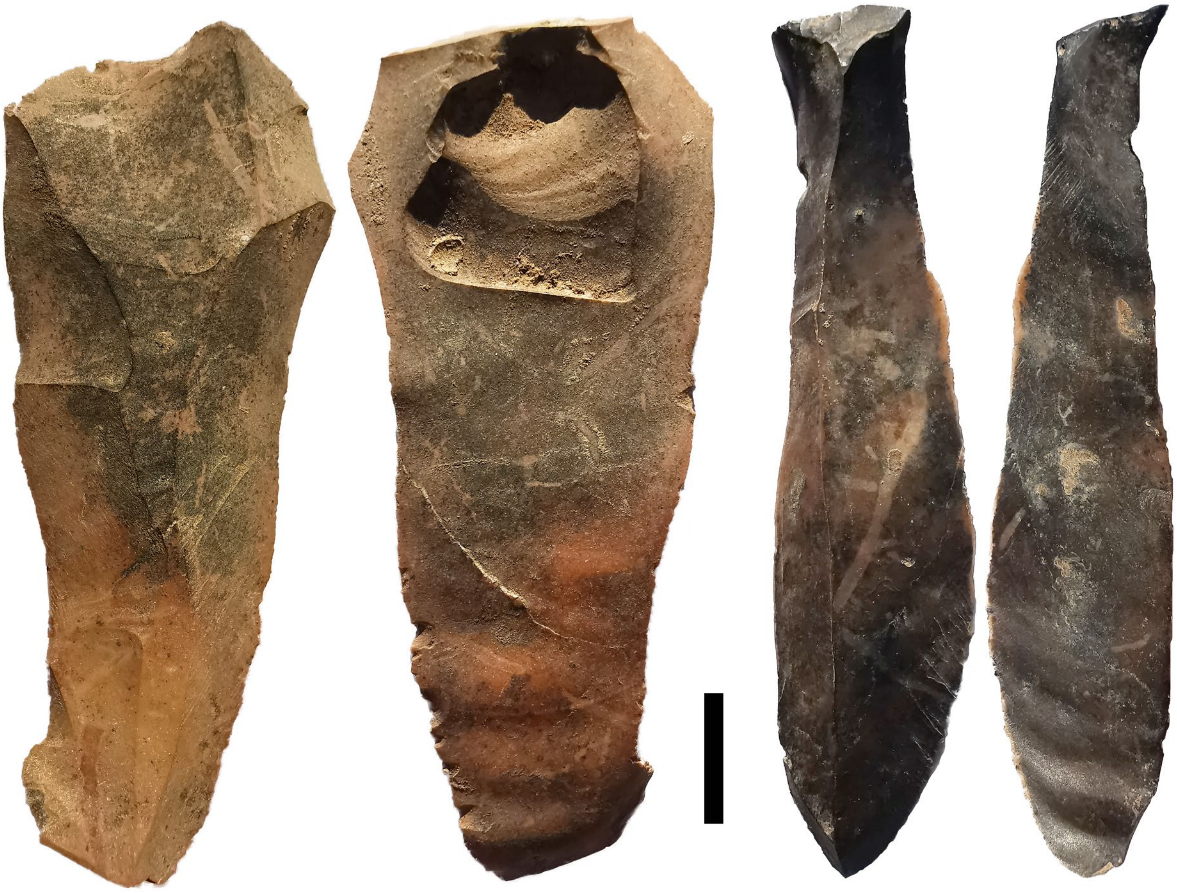

Supplementary Figure 8. *Examples of blades from Laili layers 20 (left) and 17 (right). Note the overhang removal on the piece on the right. Scale bar is 1 cm long.*

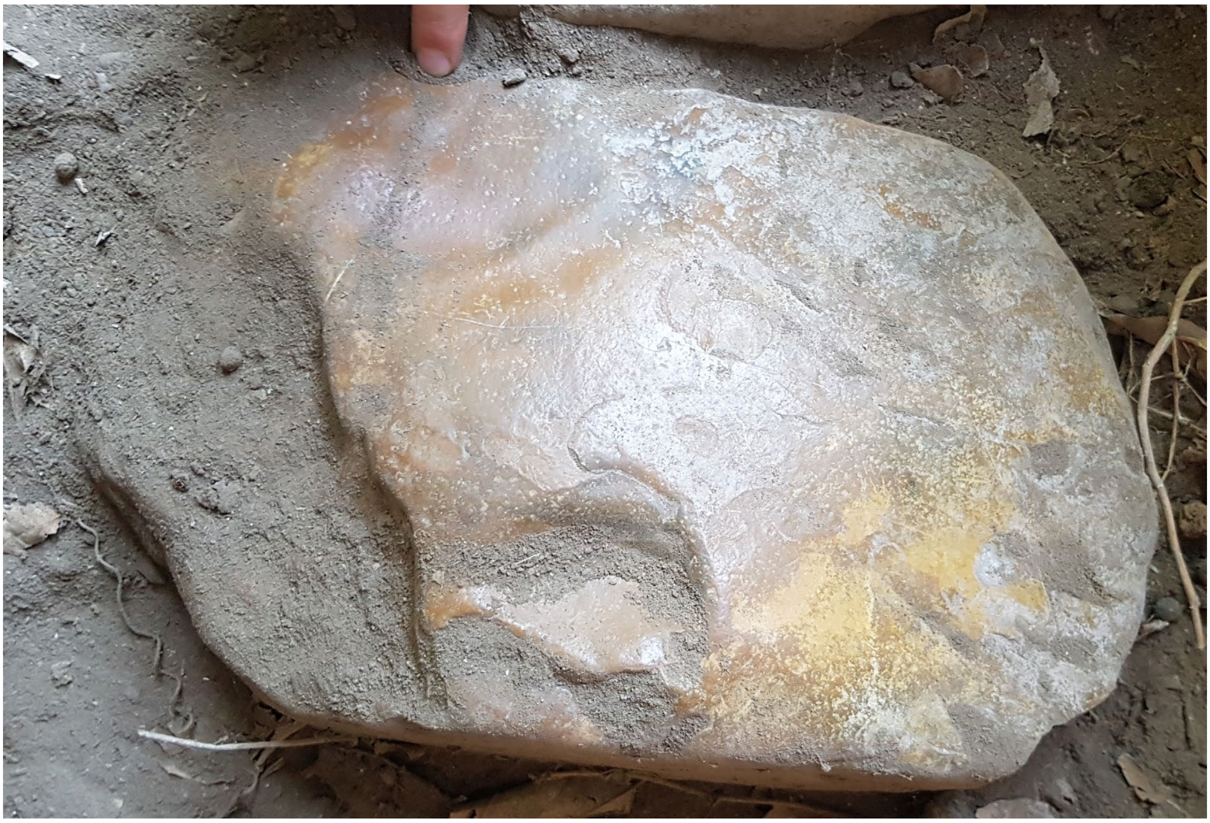

Supplementary Figure 9. *Large chert cobble on the surface at Laili. Note the finger scale at the top of the image (17 mm wide at the base of the nail).*

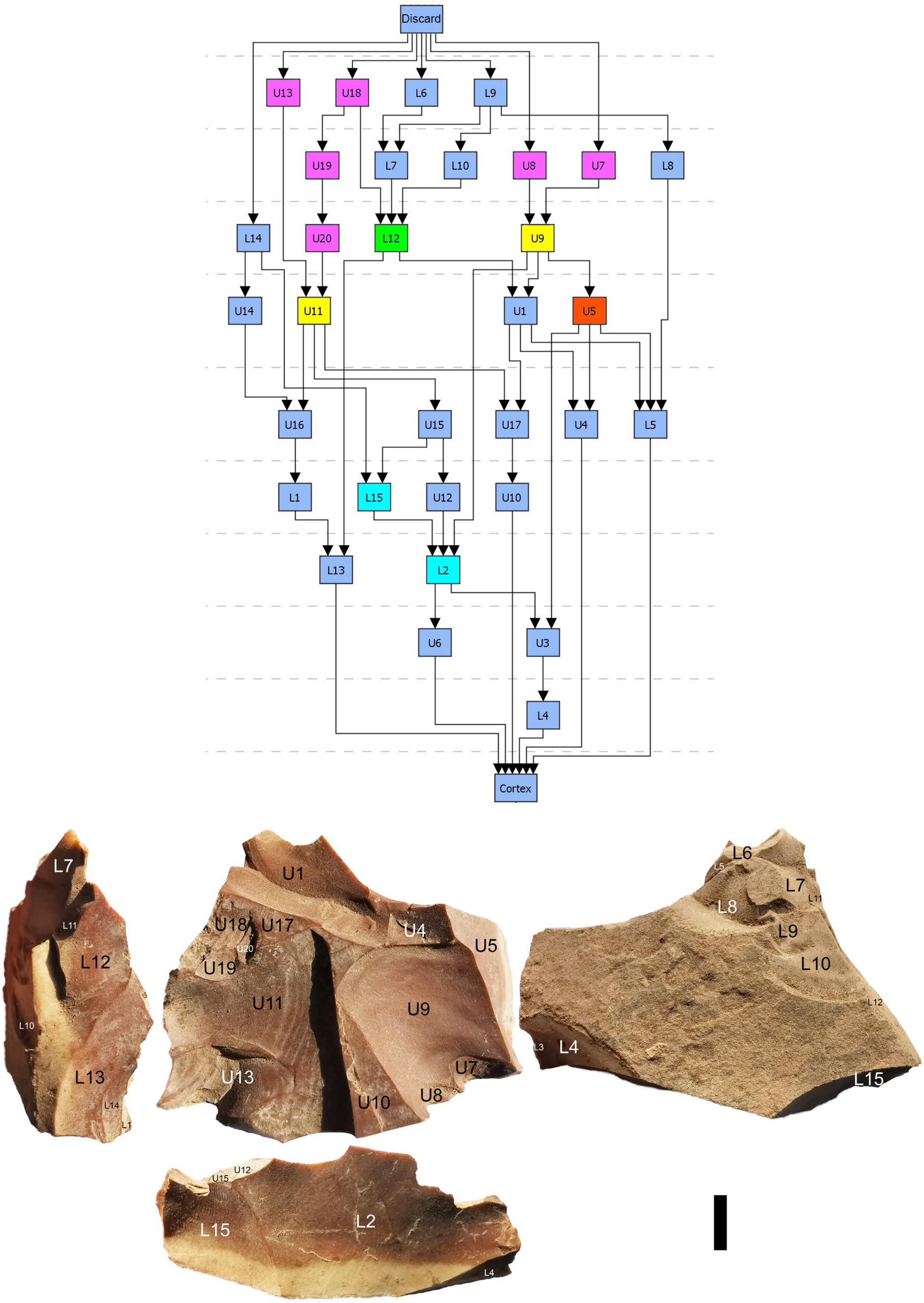

Supplementary Figure 10. *Harris Matrix showing the life history of a core and multiple notch from Laili layer 20. Cyan shows platform creating flake scars, orange shows a convexity creating flake scar, yellow shows preferential flake scars, green shows a platform rejuvenation flake scar, and pink shows notch creating flake scars. Scale bar is 1 cm long.*

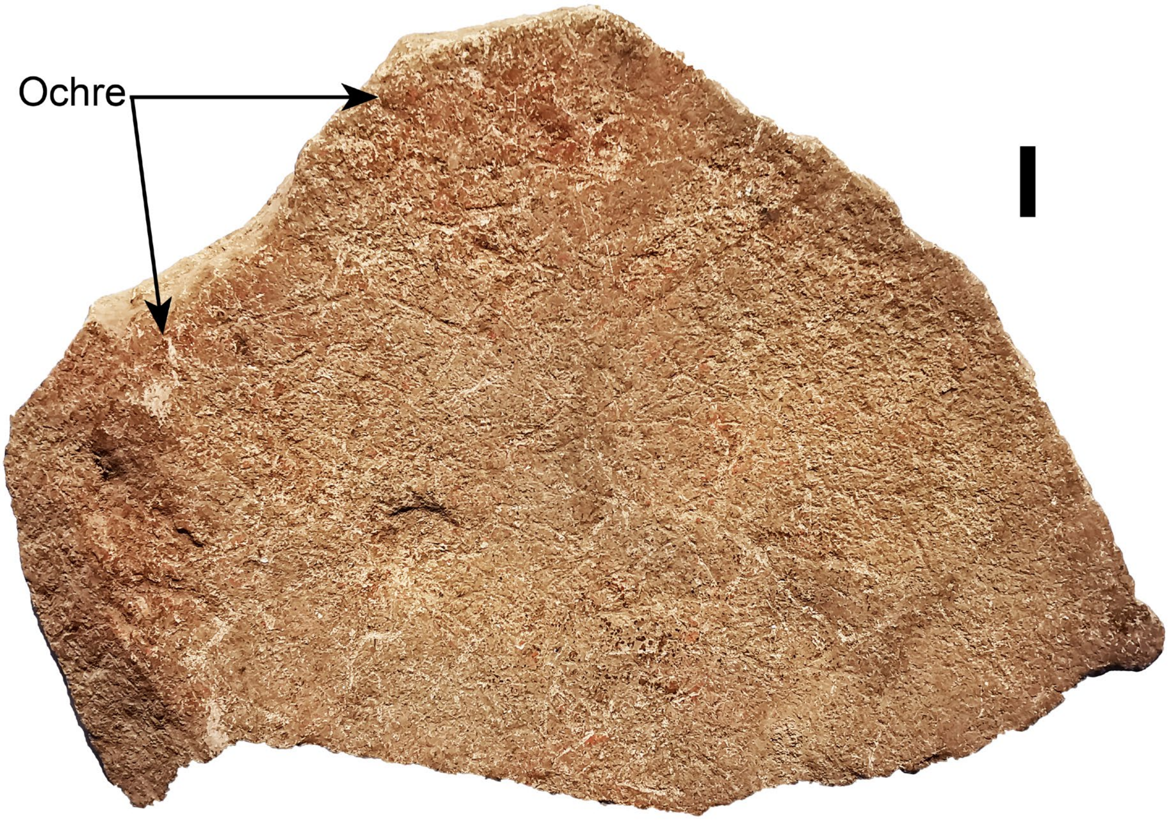

Supplementary Figure 11. *An ochre covered grinding stone from Layer 19. Scale bar is 1 cm long.*

L19-8

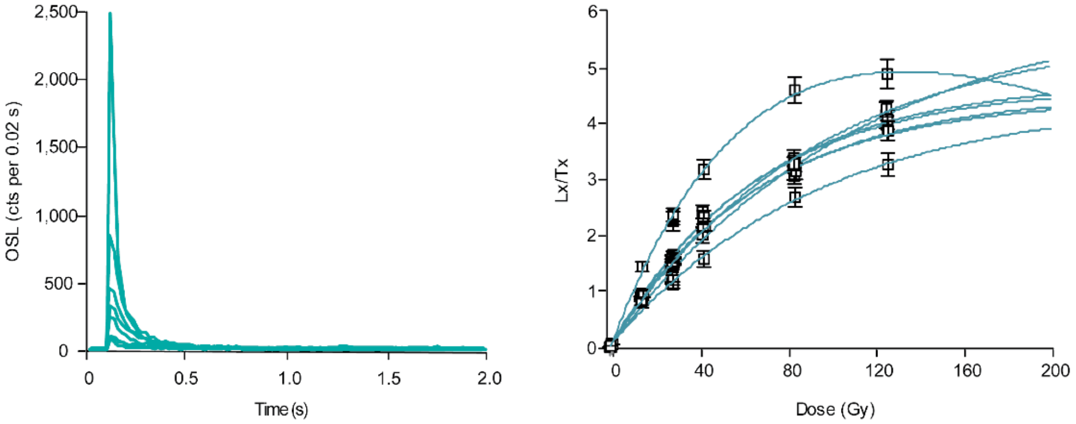

L19-11A

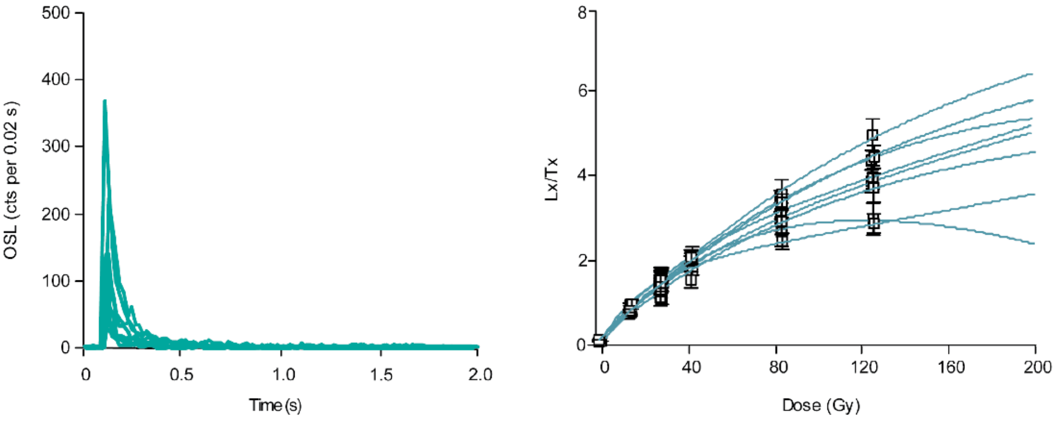

Supplementary Figure 11. *Representative OSL decay curves and their corresponding dose response curves for a selection of grains from two samples from near the top (L19-8) and bottom (L19-11A) of the stratigraphic profile.*

A

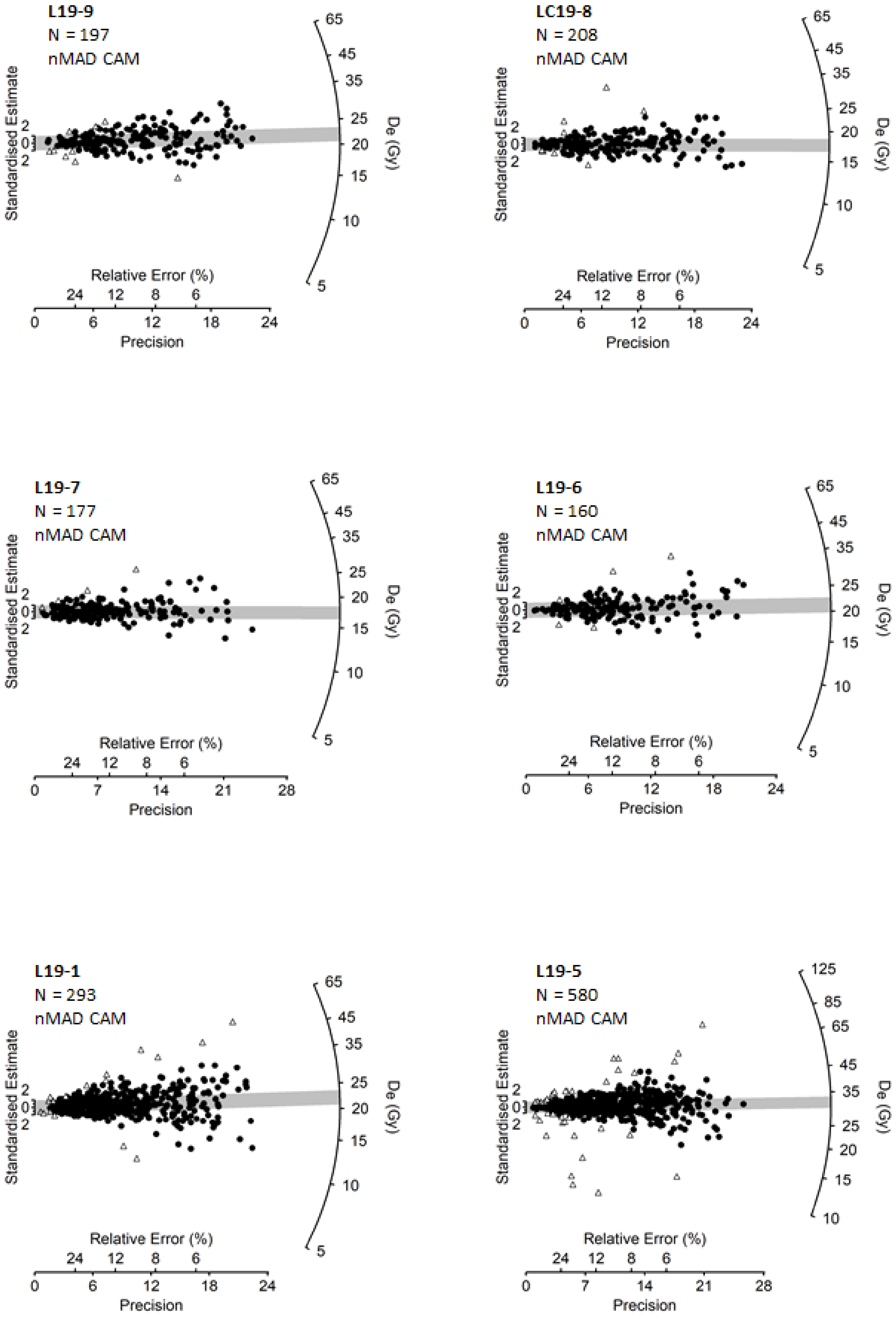
L19-9
N = 197
nMAD CAM
LC19-8
N = 208
nMAD CAM
L19-7
N = 177
nMAD CAM
L19-6
N = 160
nMAD CAM
L19-1
N = 293
nMAD CAM
L19-5
N = 580
nMAD CAM
Standardised Estimate
De (Gy)
Relative Error (%)
Precision

B

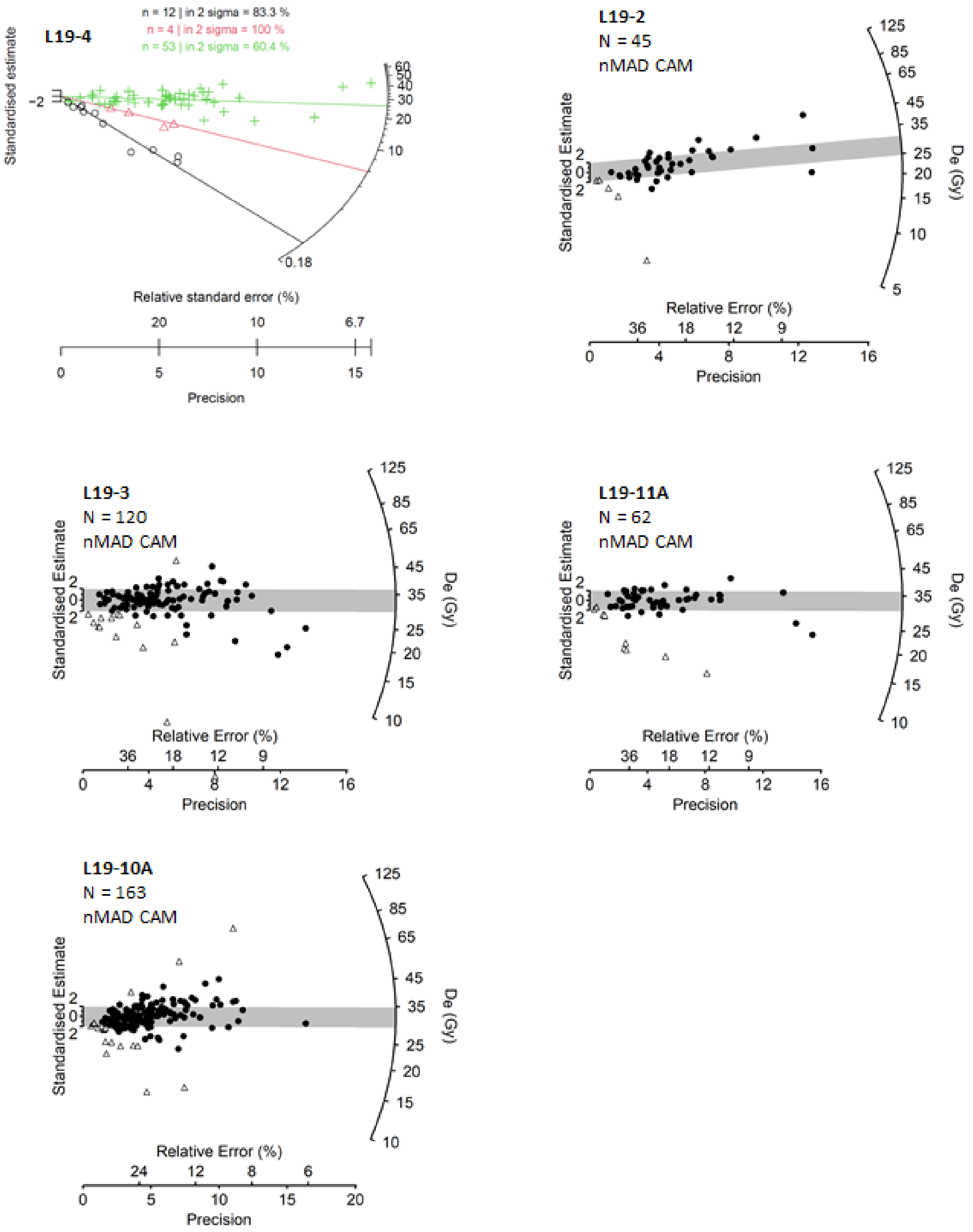

Supplementary Figure 12. *Radial plots for the eleven samples measured in this study. 12A shows samples from cultural layers (L19-9 is from Layer 9, LC19-8 is from Layer 13, L19-7 is from pit fill 15.2, L19-6 is from pit fill 15.3, L19-1 is from pit fill 15.3, and L19-5 is from Layer 19). 12B shows samples from the sterile Layer 21 (L19-4 is from 405 cm below the Holocene surface (preserved as breccia on the rockshelter wall), L19-2 is from 415 cm below the Holocene surface, L19-3 is from 460 cm below the Holocene surface, L19-11A is*

from 475 cm below the Holocene surface, and L19-10A is from 505 cm below the Holocene surface). The grey band is centred on the D_e value determined using the central age model after removal of outliers (shown as open triangles) identified using nMAD. Negative values are not shown. For L19-4 the main D_e component (as determined by the FMM and used for age estimation) is shown with green crosses, and the minor D_e components are shown in red triangles and black circles.

A

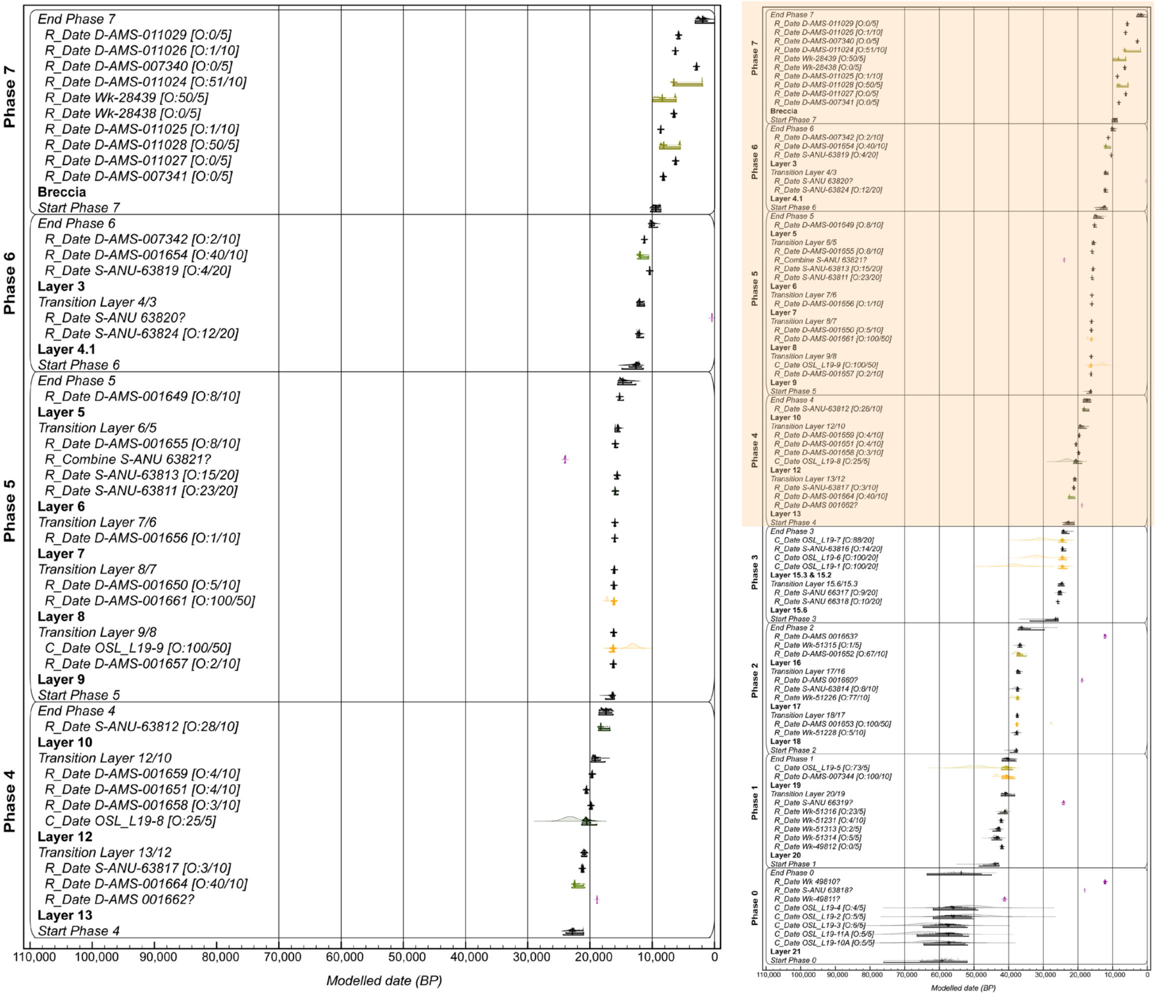

B

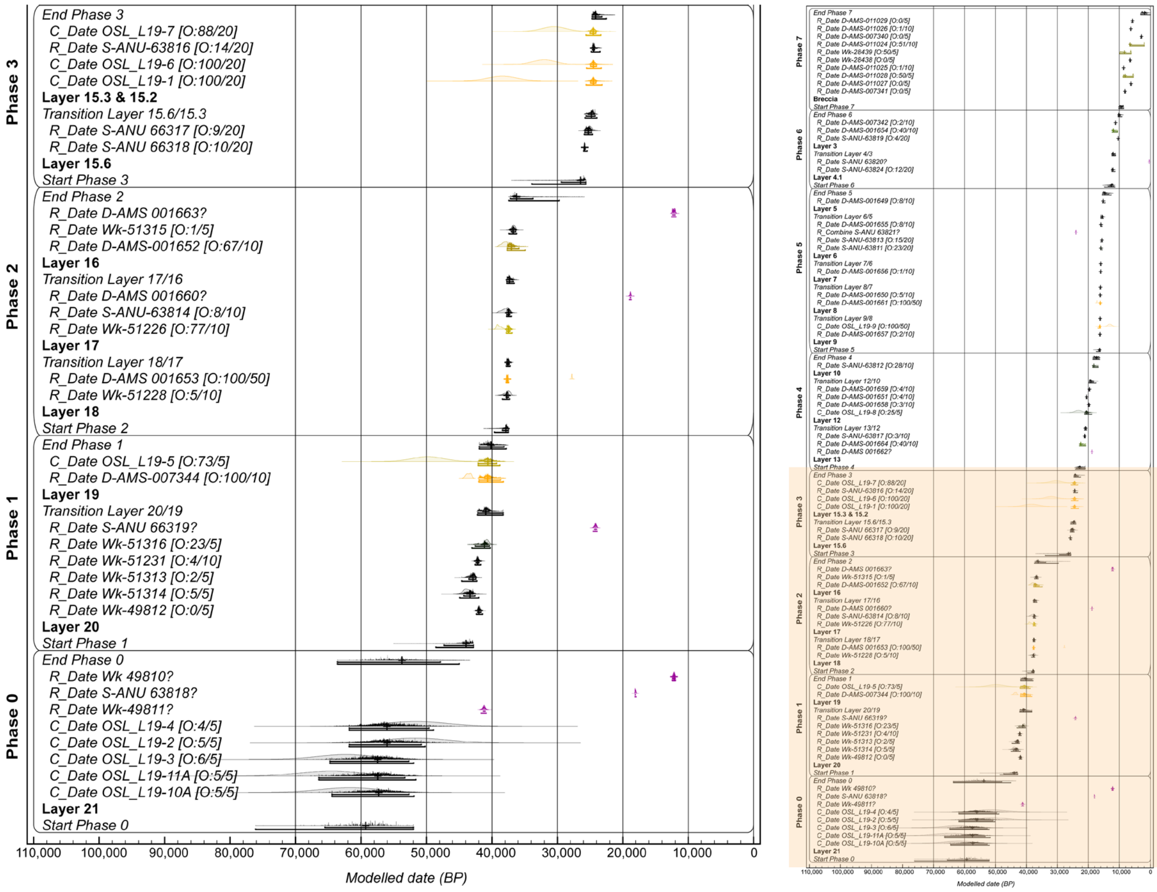

Supplementary Figure 13. *Bayesian date model for the Laili archaeological sequence, (A = upper sequence (MIS2); B = lower sequence (MIS3) (left) and overall model (right). Outliers greater than 65% probability are coloured yellow-brown and shaded from darker to lighter in increasing outlier probability. Extreme outliers that were excluded from the model are shown in purple. The brackets beneath the distributions represent the 68.3% and 95.4% probability ranges, respectively. Prior and posterior outlier probabilities are given in brackets following the sample name in the form [O: posterior/prior].*

Supplementary Tables

Supplementary Table 1. *Breakdown of mollusc Minimum Number of Individuals by taxa and layer for Laili Squares D and E.*

Taxa	16	17	18	19	20	21
Lunella cinerea	4	7	13	8	22	0
Other Turbinidae	11	15	7	6	18	2
Nerita spp.	5	11	8	4	8	1
Acanthopleura sp.	4	6	8	11	21	2
Stenomelania sp.	4	6	6	4	4	1
Other aquatic	9	11	8	4	16	0
Total aquatic	37 (8%)	56 (9%)	50 (6%)	37 (7%)	89 (24%)	6 (2%)
Camaenidae cf. *Chloritis*	448	560	731	476	279	369
Amphidromus sp.	6	7	17	5	5	0
Unknown landsnail	1	1	8	1	2	0
Total landsnail	455 (92%)	568 (91%)	756 (94%)	482 (93%)	286 (76%)	369 (98%)
Grand Total	492 (100%)	624 (100%)	790 (100%)	519 (100%)	375 (100%)	375 (100%)

Other aquatic includes Haliotis *sp*., Cellana radiata, Rochia niltoica, Tectus pyramis, Trochus maculatis, Trochus *sp.,* Monodonta canalifera, Septaria *cf*. luzonica, Terebralia *spp*., Strombus sp., Mancinella alouina, Pictocolumbella *cf*. ocellata, Chicoreus capucinus, Mitra *sp.*, Cymbiola verspetilio, Conus *sp.*, Conus marmoreus*,* Austriella *cf.* corrugata, Tridacna *sp.*, and Vasticardium *sp.*

Supplementary Table 2. *Breakdown of tetrapod Number of Individual Specimens (NISP) by taxa and layer for Laili Square C. Percentages are for non-murid identifiable specimens only by layer.*

Taxa	16	17	18	19	20	21
Teleostei (ray-finned fish)	19 (10%)	54 (12%)	94 (24%)	53 (36%)	93 (63%)	11 (3%)
Anura (frogs)	5 (2%)	26 (6%)	19 (5%)	6 (4%)	4 (3%)	77 (19%)
Lacertilia (lizards)	23 (12%)	75 (17%)	57 (15%)	10 (7%)	5 (3%)	145 (37%)
Serpentes (snakes)	8 (4%)	8 (2%)	6 (2%)	6 (4%)	12 (8%)	38 (10%)
Turtle	0	0	0	1 (1%)	0	0
Varanid	0	0	0	0	0	2 (<1%)
Aves (birds)	121 (62%)	238 (54%)	186 (47%)	69 (47%)	24 (16%)	71 (18%)
Microbats	20 (10%)	38 (9%)	29 (7%)	2 (1%)	9 (6%)	48 (12%)
Pteropodidae (fruit bats)	0	1 (<1%)	2 (<1%)	0	0	4 (1%)
Total non-murid	196 (100%)	440 (100%)	393 (100%)	147 (100%)	147 (100%)	396 (100%)
Small Murid	549	1016	767	348	205	5706
Giant Murid	51	75	57	9	16	496
Murid to non-murid ratio	3.06	2.48	2.1	2.43	1.5	15.66
Undifferentiated	329	914	693	249	172	4360

Supplementary Table 3. *Breakdown of complete flake (including retouched flakes) platform types and dorsal scar patterns by layer for Laili Squares C, D, and E.*

	**Layer**				
**Platform type**	**16**	**17**	**18**	**19**	**20**
Cortical	8 (4%)	13 (4%)	10 (4%)	3 (2%)	10 (5%)
Crushed	43 (22%)	81 (22%)	44 (17%)	26 (20%)	40 (22%)
Single	82 (42%)	182 (50%)	129 (51%)	52 (40%)	72 (39%)
Focalized	40 (20%)	49 (14%)	34 (13%)	25 (19%)	35 (19%)
Dihedral	15 (8%)	22 (6%)	16 (6%)	5 (4%)	12 (6%)
Multiple	9 (4%)	14 (4%)	22 (9%)	20 (15%)	17 (9%)
**Dorsal scar pattern**					
Cortical	2 (1%)	1 (>1%)	2 (1%)	2 (2%)	0
Proximal	121 (61%)	219 (61%)	119 (47%)	51 (39%)	90 (48%)
Orthogonal	17 (9%)	37 (10%)	46 (18%)	12 (9%)	25 (13%)
Bidirectional	22 (11%)	33 (9%)	37 (15%)	27 (20%)	31 (16%)
Radial	13 (6%)	14 (4%)	20 (8%)	12 (9%)	17 (9%)
Janus	17 (9%)	44 (12%)	16 (6%)	21 (16%)	21 (11%)
Redirecting	6 (3%)	13 (4%)	12 (5%)	7 (5%)	5 (3%)

Supplementary Table 4. *Retouched and heat damaged lithics by layer for Laili Squares C, D, and E.*

	**Layer**					
**Retouched type**	**16**	**17**	**18**	**19**	**20**	**Total**
Burin	0	1	0	0	2	3
Notch	2	4	4	4	10	24
Scraper	0	0	4	0	4	8
Ventral	0	1	2	0	1	4
Miscellaneous	0	0	0	3	3	6
Total	2	6	10	7	20	45
Percent of all complete lithics	1	1.6	3.8	5.2	10.4	4.1
**Heat damage**						
Number of lithics	16	26	31	10	23	
Percent of all complete lithics	8.1	7.3	12.4	8	12	

Supplementary Table 5. *Sample field and lab codes, layer, and depth below present and Holocene surface.*

Field Code	CABAH code	Layer	Depth below present surface (cm)	Depth below Holocene surface (cm)
L19-9	CABAH-531	9	50	250
L19-8	CABAH-530	12	95	295
L19-7	CABAH-529	Pit (15.2)	145	345
L19-5	CABAH-527	19	185	385
L19-1	CABAH-523	Pit (15.3)	197	397
L19-6	CABAH-528	Pit (15.3)	200	400
L19-4	CABAH-526	21	205	405
L19-2	CABAH-524	21	215	415
L19-3	CABAH-525	21	260	460
L19-11A-B	CABAH-534–5	21	275	475
L19-10A-B	CABAH-532–3	21	305	505

Supplementary Table 6. *Single-grain SAR measurement procedure used in this study.*

Step	Treatment
1	Give regenerative dose, D_i [a]
2	Preheat at 260 °C for 10 s
3	Single-grain green laser stimulation at 125 °C for 2 s (L_n or L_x signal observed)
4	Give test dose, D_t
5	Preheat at 160 °C for 5 s
6	Single-grain green laser stimulation at 125 °C for 2 s (T_n or T_x signal observed)
7	Return to step 1 [b]

[a] For the natural samples, D_i = 0 Gy. The OSL signals for the natural dose and corresponding test dose are denoted L_n and T_n respectively, and the OSL signals for the regenerative doses and corresponding test doses are denoted L_x and T_x, respectively. The SAR sequence was repeated for several regenerative doses, including a zero dose and a duplicate dose, to monitor the extent of recuperation and determine the recycling ratio, respectively.

[b] A final (triplicate) regenerative dose cycle was included at the end of the sequence to check for feldspar contamination of individual quartz grains based on their OSL IR depletion ratios.

Supplementary Table 7. *Number of individual quartz grains measured, rejected and accepted for each, together with the reasons for grain rejection (see text for reference to numbers. Samples are listed in the stratigraphic order of the layer from which they were collected (Layers 9–19, brown), and in depth order for Layer 21 (cream).*

Sample	Grain size (µm)	No. of grains measured	Rejection criteria							Sum of grains rejected	No. of grains accepted	Negative D_e values
			1	2	3	4	5	6	7			
L19-9	125–90	500	165	2	77	21	37	1	0	303	197	0
L19-8	125–90	500	163	2	47	21	58	0	1	292	208	0
L19-7	125–90	500	205	3	52	20	42	1	0	323	177	0
L19-6	125–90	500	204	4	64	19	48	1	0	340	160	0
L19-1	125–90	1500	647	8	185	62	101	2	0	1005	495	2
L19-5	125–90	1400	389	6	205	81	138	0	0	819	581	1
L19-4	125–90	2000	1715	4	25	49	123	0	1	1917	83	14
L19-2	125–90	1500	1353	4	24	23	51	0	0	1455	45	0
L19-3	125–90	3600	3137	13	47	91	184	1	0	3474	126	6
L19-11A	125–90	2000	1733	1	28	53	112	1	0	1928	72	10
L19-10A	125–90	2900	2320	5	60	53	299	0	0	2737	163	0

Supplementary Table 8. *Results of dose recovery tests on L19-2 using three preheat (PH) combinations.*

Sample	PH_1	PH_2	Number of grains measured/accepted	Measured/given dose ratio	OD (%)
L19-2	220 °C/10 s	160 °C/5 s	1000/24	0.90 ± 0.05	9 ± 1%
L19-2	260 °C/10 s	160 °C/5 s	1000/24	0.96 ± 0.06	2 ± 1%
L19-2	260 °C/10 s	220 °C/5 s	1000/19	0.88 ± 0.07	19 ± 1%

Supplementary Table 9. *Dose rate data, equivalent dose (D_e) and overdispersion (OD) values, and ages. Samples are listed in the stratigraphic order of the layer from which they were collected (Layers 9–19, brown), and in depth order for Layer 21 (cream).*

Sample	Layer	Water (%)	External dose rate (Gy/ka)				D_e (Gy)	OD (%)	Statistical Model	Optical age (ka)
			Beta	Gamma	Cosmic	Total				
L19-9	9	15 ± 4 (13)	1.13 ± 0.06	0.50 ± 0.06	0.023	1.67 ± 0.06	21.9 ± 0.4	27 ± 2 (24)	nMAD CAM	13.1 ± 0.6
L19-8	13	10 ± 3 (10)	0.44 ± 0.02	0.27 ± 0.01	0.022	0.77 ± 0.03	17.7 ± 0.4	32 ± 2 (24)	nMAD CAM	23.2 ± 1.1
L19-7	Pit: 15.2	20 ± 5 (19)	0.34 ± 0.02	0.18 ± 0.01	0.023	0.57 ± 0.03	17.5 ± 0.4	31 ± 2 (27)	nMAD CAM	30.5 ± 1.8
L19-6	Pit: 15.3	20 ± 5 (19)	0.41 ± 0.03	0.20 ± 0.01	0.023	0.66 ± 0.03	21.1 ± 0.5	28 ± 2 (24)	nMAD CAM	32.0 ± 1.8
L19-1	Pit: 15.3	20 ± 5 (19)	0.39 ± 0.03	0.16 ± 0.01	0.020	0.59 ± 0.03	21.9 ± 0.3	32 ± 1 (27)	nMAD CAM	38.4 ± 2.2
L19-5	19	15 ± 4 (12)	0.40 ± 0.02	0.19 ± 0.01	0.021	0.64 ± 0.03	31.7 ± 0.4	48 ± 2 (25)	nMAD CAM	49.8 ± 2.5
L19-4	21	20 ± 5 (18)	0.29 ± 0.02	0.17 ± 0.01	0.021	0.51 ± 0.03	26.3 ± 1.9	138 ± 13 (39)	FMM (74%)	51.6 ± 4.7
L19-2	21	20 ± 5 (13)	0.32 ± 0.02	0.16 ± 0.01	0.020	0.53 ± 0.03	27.5 ± 2.0	107 ± 12 (39)	nMAD CAM	51.7 ± 4.8
L19-3	21	20 ± 5 (23)	0.34 ± 0.02	0.14 ± 0.01	0.020	0.53 ± 0.03	33.0 ± 1.4	84 ± 6 (35)	nMAD CAM	62.3 ± 4.3
L19-11A	21	20 ± 5 (22)	0.30 ± 0.02	0.17 ± 0.01	0.020	0.52 ± 0.03	33.4 ± 1.7	97 ± 10 (28)	nMAD CAM	64.5 ± 4.9
L19-10A	21	30 ± 8 (30)	0.31 ± 0.03	0.16 ± 0.01	0.020	0.52 ± 0.03	32.2 ± 1.2	67 ± 4 (37)	nMAD CAM	61.7 ± 4.5
