# Supplementary material for: Abrupt onset of intensive human occupation 44,000 years ago on the threshold of Sahul
Source: Nat Commun. 2024 May 22;15:4193. doi: 10.1038/s41467-024-48395-x (PMC11111772; doi:10.1038/s41467-024-48395-x)
Supplement: Supplementary file 3 — Description of Additional Supplementary Files [file 41467_2024_48395_MOESM3_ESM.pdf]

Description of Additional Supplementary Files

Supplementary Data 1: Details of dating sample collection, radiocarbon date raw information, and Bayesian modelled ages of radiocarbon and OSL dates

Supplementary Code 1: Laili oxcal Bayesian Model: Bayesian model code for OxCal
