# Supplementary material for: Abrupt onset of intensive human occupation 44,000 years ago on the threshold of Sahul
Source: Nat Commun. 2024 May 22;15:4193. doi: 10.1038/s41467-024-48395-x (PMC11111772; doi:10.1038/s41467-024-48395-x)
Supplement: Supplementary file 6 — Reporting Summary [file 41467_2024_48395_MOESM6_ESM.pdf]

nature portfolio

Corresponding author(s): Ceri Shipton, Mike Morley, Shimona Kealy

Last updated by author(s): 18/4/24

Reporting Summary

Nature Portfolio wishes to improve the reproducibility of the work that we publish. This form provides structure for consistency and transparency in reporting. For further information on Nature Portfolio policies, see our Editorial Policies and the Editorial Policy Checklist.

Statistics

For all statistical analyses, confirm that the following items are present in the figure legend, table legend, main text, or Methods section.

n/a	Confirmed	
☐	☒	The exact sample size (*n*) for each experimental group/condition, given as a discrete number and unit of measurement
☐	☒	A statement on whether measurements were taken from distinct samples or whether the same sample was measured repeatedly
☐	☒	The statistical test(s) used AND whether they are one- or two-sided<br>*Only common tests should be described solely by name; describe more complex techniques in the Methods section.*
☒	☐	A description of all covariates tested
☒	☐	A description of any assumptions or corrections, such as tests of normality and adjustment for multiple comparisons
☐	☒	A full description of the statistical parameters including central tendency (e.g. means) or other basic estimates (e.g. regression coefficient) AND variation (e.g. standard deviation) or associated estimates of uncertainty (e.g. confidence intervals)
☐	☒	For null hypothesis testing, the test statistic (e.g. *F*, *t*, *r*) with confidence intervals, effect sizes, degrees of freedom and *P* value noted<br>*Give P values as exact values whenever suitable.*
☐	☒	For Bayesian analysis, information on the choice of priors and Markov chain Monte Carlo settings
☒	☐	For hierarchical and complex designs, identification of the appropriate level for tests and full reporting of outcomes
☒	☐	Estimates of effect sizes (e.g. Cohen's *d*, Pearson's *r*), indicating how they were calculated

Our web collection on statistics for biologists contains articles on many of the points above.

Software and code

Policy information about availability of computer code

Data collection	Microsoft Excel v.365
Data analysis	Microsoft Excel for Figure 4 and Supplementary Figures 6 and 7; IBM SPSS v.29 for Figure 5; Imagination Computer Services Harris Matrix Composer v.2 for Supplementary Figures 2 and 10; OxCal v.4.4 for Supplementary Figure 13

For manuscripts utilizing custom algorithms or software that are central to the research but not yet described in published literature, software must be made available to editors and reviewers. We strongly encourage code deposition in a community repository (e.g. GitHub). See the Nature Portfolio guidelines for submitting code & software for further information.

Data

Policy information about availability of data

All manuscripts must include a data availability statement. This statement should provide the following information, where applicable:

- Accession codes, unique identifiers, or web links for publicly available datasets
- A description of any restrictions on data availability
- For clinical datasets or third party data, please ensure that the statement adheres to our policy

All data used in this study are available in the Supplementary Information, the Supplementary Data file, or the Source Data file. Finds were labelled with the trench code LA19, the square (D/C/E), and the excavation unit (1-44). Finds are temporarily housed at the Australian National University and University College London, but long-term curation will be at the Timor-Leste Museum and Cultural Centre.

Research involving human participants, their data, or biological material

Policy information about studies with human participants or human data. See also policy information about sex, gender (identity/presentation), and sexual orientation and race, ethnicity and racism.

Reporting on sex and gender	N/A
Reporting on race, ethnicity, or other socially relevant groupings	N/A
Population characteristics	N/A
Recruitment	N/A
Ethics oversight	N/A

Note that full information on the approval of the study protocol must also be provided in the manuscript.

Field-specific reporting

Please select the one below that is the best fit for your research. If you are not sure, read the appropriate sections before making your selection.

☐ Life sciences ☐ Behavioural & social sciences ☒ Ecological, evolutionary & environmental sciences

For a reference copy of the document with all sections, see nature.com/documents/nr-reporting-summary-flat.pdf

Ecological, evolutionary & environmental sciences study design

All studies must disclose on these points even when the disclosure is negative.

Study description	Archaeology, a mixture of quantitative and qualitative methods
Research sample	3 m square of sediment excavated to a depth of 2.5 m. A location in the middle of the shleter was chosen to give the most representative sample.
Sampling strategy	Wet-sieving entire sample through 1 mm mesh. All stone artefacts and identifable fish bone was examined. Molluscs from two square metres and tetrapod fauna from one square metre was examined due to time constraints of the specialists. These square metres were chosen to give the best representation of MIS3 layers (i.e. not cut into by large MIS2 pits) in the case of squares C and D, with the molluscs from square E examined so that we can invetigate the purpose of the large pits in future work.
Data collection	Pro forma excavation context sheets in the field. Calliper measurements of stone artefacts in the laboratory. Visual assessment against comparative collections of fauna in the laboratory. Adelaide Petrographics manufactured the micromorphology thin sections.
Timing and spatial scale	Excavation in June 2019. Other data collection continued until January 2023.
Data exclusions	Material from burrows identified during excavation was discarded as this has lost its stratigraphic integrity. Burrows were identified in 23 out of 130 excavation contexts.
Reproducibility	Intra-observer reliability of the micropmorph slide interpretation was achieved by re-counting areas of selected thin sections.
Randomization	Group allocation was based on stratigraphic position.
Blinding	Blinding was not used as most researchers were involved in the fieldwork as well as the laboratory data collection.

Did the study involve field work? ☒ Yes ☐ No

Field work, collection and transport

Field conditions	It was warm and dry in Laleia over the excavation period.
Location	-8.540118, 126.163390
Access & import/export	The excavation and export of finds was carried out with permission of the Timor-Leste Ministry of Higher Education, Science and Culture, under permit numbers: 71- and 136- /DGAC-SEAC/MESCC/VI2019, issued on 5th June 2019.

Disturbance | The excavation was filled in with sand-bagged sediment upon completion.

Reporting for specific materials, systems and methods

We require information from authors about some types of materials, experimental systems and methods used in many studies. Here, indicate whether each material, system or method listed is relevant to your study. If you are not sure if a list item applies to your research, read the appropriate section before selecting a response.

Materials & experimental systems

n/a	Involved in the study
☒	☐ Antibodies
☒	☐ Eukaryotic cell lines
☐	☒ Palaeontology and archaeology
☒	☐ Animals and other organisms
☒	☐ Clinical data
☒	☐ Dual use research of concern
☒	☐ Plants

Methods

n/a	Involved in the study
☒	☐ ChIP-seq
☒	☐ Flow cytometry
☒	☐ MRI-based neuroimaging

Palaeontology and Archaeology

Specimen provenance: Timor Leste, Ministry of Tourism, Arts and Culture, permit number: 136/DGAC-SEAC/MESCC/VI/2019

Specimen deposition: Finds are temporarily housed at the Australian National University and University College London, but long-term curation will be at the Timor-Leste Museum and Cultural Centre.

Dating methods: Dating methods are described in detail in the Supplementary Information and Supplementary Dataset 1

☒ Tick this box to confirm that the raw and calibrated dates are available in the paper or in Supplementary Information.

Ethics oversight: Timor Leste, Ministry of Tourism, Arts and Culture, permit number: 71/DGAC-SEAC/MESCC/VI/2019

Note that full information on the approval of the study protocol must also be provided in the manuscript.
